# No Value of Routine Brain Computed Tomography 6 Weeks after Evacuation of Chronic Subdural Hematoma

**DOI:** 10.1055/s-0037-1607215

**Published:** 2017-11-27

**Authors:** Christian Bonde Pedersen, Filippa Sundbye, Frantz Rom Poulsen

**Affiliations:** 1 Department of Neurosurgery, Odense University Hospital, Odense, Denmark; 2 Clinical Institute, University of Southern Denmark, Odense, Denmark

**Keywords:** chronic subdural hematoma, postoperative CT scan, evacuation, recurrence

## Abstract

**Background** The aim of this study was to evaluate the value of planned control postoperative brain computed tomography (CT) scan performed 4 to 6 weeks after the evacuation of chronic subdural hematoma.

**Materials and Methods** This retrospective study examined 202 patients who during a 2-year period from 2011 and 2012 underwent surgical treatment for chronic subdural hematoma (CSDH). Information on patient age, sex, alcohol consumption, anticoagulant/antiplatelet treatment, history of head trauma, Glasgow coma scale (GCS), neurological symptoms, laterality of CSDH, and surgical technique was retrieved from patient charts.

**Results** Overall, 27 out of 202 patients had a recurrence of CSDH and re-evacuation of the hematoma was performed. In all patients recurrence of neurological symptoms preceded the planned postoperative control brain CT 4 to 6 weeks after primary surgery.

**Conclusion** Routinely postoperative control brain CT scan 4 to 6 weeks after the evacuation of a CSDH has no clinical value.

Chronic subdural hematoma (CSDH) is a common neurosurgical condition, especially among elderly. The incidence is 58/100,000 persons per year in people over the age of 70. 1 Predisposing factors for CSDH includes cerebral atrophy, use of anticoagulants, and chronic alcohol use. CSDH develops due to head trauma, although this can only be confirmed in 50% of the patients. 2 3 The diagnosis is most often based on a brain computed tomography (CT) scan. Symptoms indicative of CSDH include headache, cognitive symptoms, and focal neurological deficits, such as hemiparesis or aphasia. 2

No consensus yet exists regarding the optimal surgical technique when evacuating CSDHs. They all aim at decompressing the cerebral hemisphere and minimizing the risk of recurrence with the least morbidity and mortality. Traditionally, the methods of evacuation are either burr-hole craniostomy (BHC) or minicraniotomy often followed by drainage, either subgaleal or subdural for 24 to 48 hours postoperatively. 3 Recurrence rates of CSDH have been reported as high as 33%. 4

In our neurosurgical unit, a postoperative brain CT scan 4 to 6 weeks after the evacuation of CSDH is routinely performed. The value of this remains unclear, and the indication has mainly been based on tradition. The present study aimed at investigating the relevance of such a control brain CT 4 to 6 weeks after CSDH evacuation.

## Materials and Methods

During a year period 2011 and 2012, 297 patients underwent surgery for subdural hematoma (ICD-10 S065) at our department. Patients with an acute subdural hematoma (79), conservatively treated CSDH (15), recurrent CSDH after a primary evacuation before 2011 (2), CSDH as a complication of another surgical procedure and misdiagnosed CSDH (1) were excluded. A total of 198 patients were surgically treated for CSDH. Four patients were treated with bilateral burr holes for bilateral CSDH. Since these hematomas were bilateral, they were included in the subsequent analysis as eight CSDHs. All in all, 202 hematomas were included in the study contentiously.

For each patient sex, age, known alcohol abuse, use of anticoagulant and/or antiplatelet drugs, history of head trauma leading up to the diagnosis, Glasgow coma scale (GCS), neurological symptoms (focal and/or global), laterality, surgical technique (including type of drainage), and recurrence (defined as recurrent symptoms leading to renewed surgery) were registered retrospectively on the basis of patient charts. Incomplete data were categorized as unknown.

Accordingly, the patients were divided into two groups: Recurrence group (RG) and no recurrence group. In the RG, the time from primary surgery to the diagnosis of recurrence was registered.

Data for 25 patients were missing. Twelve patients died after surgical CSDH evacuation and before control brain CT and six were excluded due to malignant disease and no follow-up was performed. In three cases no control brain CT was prescribed, and in four cases follow-up data were lost ( Table 1 ). Due to national electronic medical record system and fixed service area of our neurosurgical unit, we checked and ensured that the patients who were lost to follow-up were not treated nor had a follow-up for CSDH elsewhere in the country.

**Table 1:** Baseline characteristics (all patients)

Patient	Sex	Age (y)	Alcohol abuse	AC and/or AP drugs	Head trauma	GCS	Symptoms	Laterality	Surgical technique
202	Female: 54	Mean: 74	Yes: 31	AC: 24	Yes: 118	15: 103	Focal: 36	Left: 92	BHC + SD: 97
	Male: 148	Median: 75	No: 157	AP: 61	No: 33	14–13: 65	Global: 69	Right: 79	BHC + SG: 99
				Both: 17	Possible: 33	12–9: 21	Both: 97	Bilateral: 31	Craniotomy + SG: 6
				None: 100	Unknown: 34	8–3: 13			

Abbreviations: AC, anticoagulant; AP, antiplatelet; BHC, burr-hole craniostomy; GCS, Glasgow coma scale; SD, subdural drainage; SG, subgaleal drainage.

Statistical analysis was performed with SPSS software version 21.0 (IBM Inc.). Univariate analysis was performed to assess the relationship between each variable and recurrence. Mann–Whitney U test was used regarding age and GCS, chi-square test for the other variables.

## Results

Patient demographics are shown in Table 1 . A total of 202 hematomas were identified (148 men and 54 women). The mean age was 74 years (range: 19–97 years). A total of 31 patients had known or a history of alcohol abuse. Overall, 24 patients used vitamin K antagonists, 61 antiplatelet drugs, and 17 both treatments. A total of 118 patients had a history of head trauma. There were 13 patients with GCS 3 to 8, 21 with GCS 9 to 12, 65 with GCS 13 to 14, and 103 with GCS 15. GCS was estimated on the basis of patients' records in 61 cases due to missing data. Focal neurological symptoms, such as hemiparesis and aphasia, were present in 36 of the patients. A total of 69 patients presented with global symptoms including headache, nausea, vomiting, and decreased level of consciousness and 97 with a combination of both. The majority ( *n* = 92) of the CSDHs were located on the left side, in line with the previous report, 5 79 were located on the right side, and 31 were bilateral. The CSDH was evacuated through a BHC followed by subdural drainage ( *n* = 97), a BHC followed by subgaleal drainage ( *n* = 99) or a minicraniotomy and subgaleal drainage ( *n* = 6).

Overall, 27 patients underwent a re-evacuation for recurrent CSDH ( Table 2 ). The time from primary evacuation till re-evacuation was 26 days (range: 2–109 days). Of the 27 patients, 22 were operated before the planned control postoperative brain CT scan was performed. Out of the remaining five patients, two had neurological symptoms when confronted with the results of the planned control brain CT scan, and they were reoperated 84 and 109 days postoperatively, respectively. Two patients had suffered from symptoms indicative of CSDH for some time but did not seek medical attention (they had dementia and severe depression, respectively) and one patient developed neurological symptoms two days before the planned postoperative brain CT scan. All of the 27 patients had neurological deficits: 7 had global symptoms, 8 had focal symptoms, and 12 had a combination of both. One had a GCS of 3 to 8, four had a GCS of 9 to 12, seven had a GCS of 13 to 14, and 15 had a GCS of 15. Due to missing data, six of the GCS scores were estimated.

**Table 2:** Recurrence group (re-evacuation)

Patient	Time a (d)	GCS	Symptoms
27	Mean: 26	15: 15	Focal: 8
	Median: 22	14–13: 7	Global: 7
	Range: 2–109	12–9: 4	Both: 12
		8–3: 1	

Abbreviation: GCS, Glasgow coma scale.

a Time from evacuation to re-evacuation.

## Discussion

In the present study, we retrospectively reviewed the time from primary surgery for CSDH to surgery for recurrence, the clinical symptoms leading to recurrent surgery and the value of planned control brain CT 4 to 6 weeks after primary surgery.

As expected, all patients with recurrent CSDH were re-evacuated due to neurological symptoms, and in 22 of 27 cases these symptoms recurred before the planned control brain CT scan was performed

Of the five patients operated after the control CT scan, only two patients sought medical assistance due to symptoms indicative of recurrence, whereas three had symptoms that could be referred to the recurrence, but these patients did not seek medical assistance. In these three cases, one patient had dementia and another from severe depression. The third patient developed symptoms just 2 days before the postoperative CT scan, which could help, explain why he waited to seek care.

The median time to re-evacuation, 22 days, is in line with other studies. 6 Mori et al reported a median time of 24.5 days. 7 The recurrence rate of 13.4% is also comparable to other studies. In a meta-analysis by Ducruet et al a recurrence rate of 11.7% after BHC was reported, 3 and Weigel et al reported a recurrence rate of 14.6% for both BHC and minicraniotomy. 8

The meta-analysis by Liu et al and the randomized trial by Santarius et al demonstrated that postoperative drainage is useful in the reduction of CSDH recurrence rate. Drainage was found to reduce recurrence by approximately 60% without an increase in morbidity and mortality. 1 9 Using irrigation during the procedure resulted in a CSDH recurrence rate of 8.0% compared with 14.1% in the irrigation-free group.

Regarding treatment modality, BHC is often first choice and minicraniotomy typically reserved for patients with either recurrent CSDH or multiple membranes on CT scan. The recurrence rate is also influenced by the aggressiveness in the removal of membranes. 10 By being too aggressive, the risk of reoperation due to postoperative hematoma is larger.

The limitation of this study is mainly the retrospective study design. There is recall bias regarding the history of head trauma, and in several cases, the patients' GCS scores were not explicitly stated in the patient files and had to be estimated.

However, even with these limitations in mind, the data does not support the routine use of planned control brain CT 4 to 6 weeks after surgical treatment for CSDH. Brain CT should only be performed if there is clinical suspicion of recurrence or in selected cases where the patients might not be able to express themselves properly, although clinical follow-up still might be as effective.

## Conclusion

Brain CT scan 4 to 6 weeks after the evacuation of CSDH should not routinely be performed. Instead, patients should be informed of the symptoms of recurrence. In selected cases, a planned control brain CT can be necessary, although in most cases clinical follow-up is just as good.
